# Genomic instability and proliferative activity as risk factors for distant metastases in breast cancer

**DOI:** 10.1038/sj.bjc.6604479

**Published:** 2008-07-29

**Authors:** L Li, K Mu, G Zhou, L Lan, G Auer, A Zetterberg

**Affiliations:** 1 Department of Pathology, Shandong University School of Medicine, 44 no., Wenhua Xi Road, Jinan, Shandong 250012, People's Republic of China; 2 Department of Oncology–Pathology, Karolinska Institutet, Cancer Center Karolinska, SE-171 76 Stockholm, Sweden

**Keywords:** ploidy, proliferative activity, distant metastases, breast cancer

## Abstract

The role of genomic instability and proliferative activity for development of distant metastases in breast cancer was analysed, and the relative contribution of these two risk factors was quantified. A detailed quantitative comparison was performed between Ki67 and cyclin A as proliferative markers. The frequency of Ki67 and cyclin A-positive cells was scored in the same microscopic areas in 428 breast tumours. The frequency of Ki67-positive cells was found to be highly correlated with the frequency of cyclin A-positive cells, and both proliferation markers were equally good to predict risk of distant metastases. The relative contribution of degree of aneuploidy and proliferative activity as risk markers for developing distant metastases was studied independently. Although increased proliferative activity in general was associated with an increased risk of developing distant metastases, ploidy level was found to be an independent and even stronger marker when considering the group of small (T1) node negative tumours. By combining proliferative activity and ploidy level, a large group of low risk breast tumours (39%) could be identified in which only a few percentage of the tumours (5%) developed distant metastases during the 9-year follow-up time period.

Numerous studies published during the last decades have clearly shown that genomic instability in terms of degree of aneuploidy (D- or A-type, Forsslund and Zetterberg, 1990; Forsslund *et al*, 1996) and chromosomal rearrangements is closely related to tumour development and tumour progression. Breast tumours of the D-type generally progressed more slowly, on the average four times, and were clinically much less aggressive than their highly aneuploid, genomically unstable counterparts of the A-type (Auer *et al*, 1980; Cornelisse *et al*, 1987; Fallenius *et al*, 1988a; Kronenwett *et al*, 2006). Chromosomal rearrangements in terms of deletions, duplications and amplifications, as studied by comparative genomic hybridization (CGH), were found to be much more frequent in the highly aneuploid breast tumours than in the diploid ones (Kallioniemi *et al*, 1994; Ried *et al*, 1995; Blegen *et al*, 2001). High-resolution microarray-based CGH data have verified and extended these findings, and identified chromosomal regions and novel specific patterns and degree of rearrangements related to aggressive tumour behaviours (Hicks *et al*, 2006). Taken together, these data clearly indicate that genomic instability is an important factor for tumour development and progression including distant metastases.

In addition to genomic instability, proliferative activity is a general property to be considered in the progression of tumours. Tumour cell proliferation has been widely investigated in breast cancer for its association with neoplastic growth, progression, metastatic potential, survival and response to chemotherapy (van Diest *et al*, 2004; Colozza *et al*, 2005; Beresford *et al*, 2006). Proliferative activity could be assessed through immunohistochemical procedures detecting proliferation-associated antigens, such as Ki67 (Gerdes, 1990), or cell-cycle-specific proteins such as cyclin A (Hunt, 1991; Sherr, 1993; Nurse, 1994). Various studies have shown that a high expression of Ki67 or cyclin A is correlated with a worse prognosis in breast cancer (Bukholm *et al*, 2001; Kuhling *et al*, 2003; Poikonen *et al*, 2005; Baldini *et al*, 2006; Ahlin *et al*, 2007; de Azambuja *et al*, 2007; Railo *et al*, 2007). However, evidence has also been obtained that the prognostic value of proliferation markers varies significantly depending on clinical characteristics of the tumour disease, for example, lymph node status (Jalava *et al*, 2006; Trere *et al*, 2006). Thus, to obtain more detailed information regarding the prognostic contribution of proliferation markers in breast cancer, patients have to be subgrouped according to clinical features, for example, tumour size and lymph node status.

The specific aim of this study was to investigate and compare in the same individual tumours, the relative influence of genomic instability and proliferative activity as risk factors for development of distant metastases in breast cancer. As one aspect of genomic instability, the degree of aneuploidy was quantified, and the tumours were separated in the two groups (A or D) with respect to ploidy level. The proliferative activity was analysed by immunohistochemistry using antibodies against Ki67 and cyclin A. An important methodological aspect of this paper was the direct quantitative comparison performed between the Ki67 analysis and the cyclin A analysis in the same tumour areas. By combining proliferative activity and ploidy level, a relatively large group of low-risk breast tumours (39%) could be identified in which only a few percentage of the tumours (5%) developed distant metastases during the 9-year follow-up time period. In the remaining 61% of the breast cancers, 35% developed distant metastases during the same follow-up time period.

## Materials and methods

### Tumour samples

This study was based on the data of 428 patients with breast cancer analysed at the department of Oncology–Pathology, Karolinska University Hospital, Solna, at the time of diagnosis (1997–1998). All histological specimens were routinely Ki67- and cyclin A-stained. In the 428 cases, 378 patients available with clinical data were followed up from diagnosis until death or survivors for at least 9 years. All tumours were classified according to the World Health Organization (1981) and graded on the basis of the recommendations of Elston and Ellis (1991). Permission to analyse the samples and correlate the results to patient data was obtained by the Ethical Committee Nord, Karolinska Institutet (Dnr 00-186). The tumour samples were fixed in 4% phosphate-buffered formaldehyde directly after operation and paraffin-embedded. From each specimen, 10 contiguous sections were prepared and used for HE staining and immunohistochemistry (thickness 4 *μ*m).

### Immunohistochemistry

The sections were deparaffinized with xylene, rehydrated through a graded alcohol series and microwaved at 500 W for 2 × 5 min in 10 mM citrate buffer (pH 6.0). After rinsing in Tris-buffered saline (TBS, pH 7.6), sections were treated with 3% hydrogen peroxide in methanol to exhaust endogenous peroxidase activity followed by normal horse serum (1 : 20 dilution) in 0.1 M PBS (pH 6.0), and then incubated overnight with the monoclonal primary antibodies diluted in 1% (wt/vol) bovine serum albumin and visualized by standard avidin–biotin–peroxidase complex technique (Vector Laboratories, Burlingame, CA, USA). Counterstaining was performed with Mayer's haematoxylin. The antibodies used were as follows: MIB-1 (antibody against the nuclear proliferation-associated antigen Ki67, Immunotech SA, Marseille, France), dilution 1 : 150; NCL-cyclin A (Cyclin A monoclonal antibody, Novocastra Laboratories Ltd, Claremont Place, Newcastle upon Tyne, UK), dilution 1 : 100.

### Evaluation of immunoreactivity scores

By comparison with the haematoxylin-and-eosin-stained sections, images of the same morphology areas expressing Ki67 and cyclin A were taken by digital camera in at least 5–14 high-power fields (10 × 40 magnification). The percentage of positive cells was measured by two experienced pathologists blinded to each other. A minimum of 1000 tumour cells were counted. Only distinct nuclear staining was accepted as a positive reaction for both markers, whereas all cells with simultaneous nuclear and cytoplasmic cyclin A staining were regarded as positive for cyclin A.

### Image cytometry

Nuclear DNA was measured by image cytometry on Feulgen-stained imprints as previously described (Auer *et al*, 1980). DNA histograms were interpreted according to a modified subjective method. The normal control cells were given the value 2*c*, denoting the normal diploid DNA content, and all tumour-cell DNA values were expressed in relation to that. The histograms were divided into two groups. Cases with a major peak near the 2*c* region (1.8*c*–2.2*c*), and <10% cells exceeding 2.2*c* were denoted diploid. DNA profiles with a stem line outside the diploid and tetraploid region and distinctly scattered DNA values exceeding the tetraploid region (3.8*c*–4.2*c*) were classified as aneuploid. Furthermore, the S-phase fraction (SPF) was measured on the basis of the DNA distribution patterns (Falkmer *et al*, 1990).

### Statistical analysis

Statistical analyses were performed using the SPSS for Windows version 11. The correlation between cyclin A, Ki67 and SPF were evaluated by Spearman's rank correlation test and the linear correlation test. Fisher's exact test was used to compare the difference between non-continuous variable. Cut-off points of Ki67 and cyclin A in patients with distant metastases were calculated by ROC curves quantitative analysis, and contribution of the risk factors to distant metastases was determined by multivariate analysis with logistic regression. *P*-value<0.05 was considered to be statistically significant.

## Results

To obtain accurate information about proliferative activity, two independent markers Ki67 and cyclin A were used, and a direct quantitative comparison between these two markers was performed. An important methodological aspect of the approach used in this paper is that the Ki67 and the cyclin A analyses were carried out on identical microscopic areas (5–14 areas in each corresponding tumour) of all of the 428 tumours. This gives particular strength to the accuracy of the quantitative data obtained on proliferative activity.

**Figure 1:**
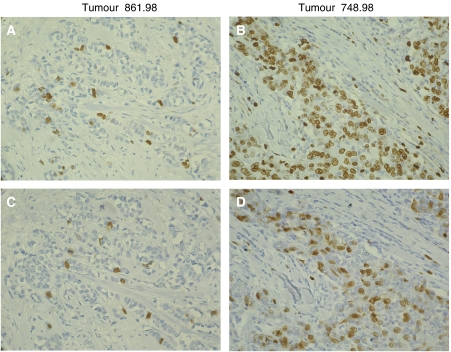
Ki67 (**A** and **B**) and cyclin A (**C** and **D**) immunostaining of the very same tumor areas of a slowly proliferating D-tumour (**A** and **C**) and a rapidly proliferating A-tumour (**B** and **D**).

Figure 1 illustrates immunostaining of two tumours, one slowly proliferating near-diploid, D-tumour (Figure 1A and C) and one rapidly proliferating clearly aneuploid, A-tumour (Figure 1B and D). The number of Ki67-positive cells is low (4%) in the D-tumour (Figure 1A) and high (40%) in the A-tumour (Figure 1B). A corresponding result is seen in the same microscopic areas of the tumours stained for cyclin A (2 and 20%, respectively; compare Figure 1A and C and Figure 1B and D). Image cytophotometric S-phase analysis of Feulgen-DNA-stained cell nuclei in the near-diploid D-tumour (Figure 1A and C) showed about 1% cells in S-phase in contrast to 15% in the A-tumour (Figure 1B and D).

**Figure 2:**
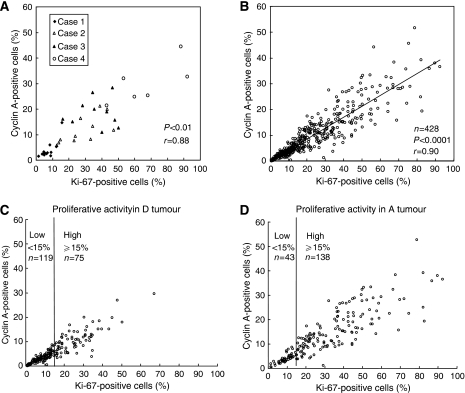
Quantitative relationship between Ki67- and cyclin A-positive cells counted in the same 5–14 randomly selected microscopic fields in four different tumours exhibiting low, intermediate and high proliferative activity (**A**), in a total of 428 tumours analysed in the same way (**B**) as well as in D-tumours (**C**) and A-tumours (**D**) performed on 375 of the 428 tumours.

Figure 2 shows the direct quantitative relationship between Ki67 and cyclin A as proliferative markers. In Figure 2A, the percentage of Ki67-positive cells is plotted against the percentage of cyclin A-positive cells counted in the same 5–14 randomly selected microscopic fields in each of four different tumours exhibiting low, intermediate and high proliferative activity (Figure 2A). The percentage of Ki67-positive cells was highly correlated (correlation coefficient 0.88) with the percentage of cyclin A-positive cells, when considering the same individual microscopic field in each of the four tumours (Figure 2A). A large variation in proliferative activity, in most cases two- to five-fold, was observed between the different microscopic fields in each tumour. This emphasises the importance of counting several different microscopic fields in each tumour to get reliable quantitative information about proliferative activity. When the analysis was performed in such a way on a set of 428 tumours, a very high correlation (correlation coefficient 0.90) was found between percentage of Ki67-positive cells and percentage of cyclin A-positive cells (Figure 2B). The data presented in Figure 2B represent the average of 5–14 randomly chosen microscopic fields in each tumour.

A comparison between D-tumours and A-tumours was performed on 375 of the 428 tumours (Figure 2C and D). A similar correlation between Ki67 and cyclin A was found in tumours of both types, a correlation coefficient of 0.90 for the D-tumours and 0.86 for the A-tumours. When comparing D- and A-tumours with respect to proliferative activity, two features could be seen. On average, the proliferative activity was twice as high in A-tumours (median value 21% Ki67-positive cells) as in D-tumours (median value 11%). However, a very large overlap in proliferative activity was found between these two groups. When using a cut-off value of 15% for Ki67-positive cells (see further Materials and Methods), the majority of the A-tumours (76%) showed high proliferative activity, whereas the majority of the D-tumours (61%) showed low proliferative activity.

Image cytophotometric S-phase determination was performed on the 375 ploidy analysed tumours. Although only about 100 cells were analysed for each tumour, which is enough for the accurate ploidy determination into D- or A-type, but probably insufficient for accurate S-phase analysis, some information about SPF could be obtained. Thus a lower, but still relatively good, correlation was found between Ki67 and SPF (correlation coefficient 0.61) and cyclin A and SPF (correlation coefficient 0.65).

**Figure 3:**
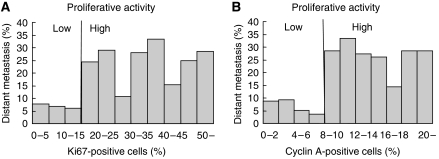
Relative risk of developing distant metastases independently of node status and tumour size when Ki67 (**A**) or cyclin A (**B**) was used as proliferative marker.

**Figure 4:**
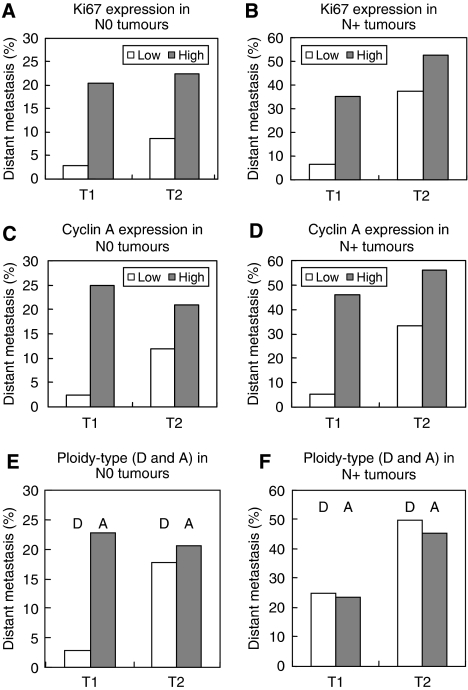
Relative risk of developing distant metastases in N0 (**A, C** and **E**) and N+ patients (**B, D** and **F**) when Ki67 (**A** and **B**), cyclin A (**C** and **D**) or ploidy type (**E** and **F**) was used as marker in T1 and T2 tumours.

A clearly increased risk of developing distant metastases is seen when the proliferative activity increases (Figure 3). The same result was obtained when either Ki67 or cyclin A was used as a proliferative marker (Figure 3A and B). Among the tumours with low proliferative activity, using a cut-off value of 15% for Ki67-positive cells and 8% for cyclin A-positive cells (see further Materials and Methods and Ahlin *et al*, 2007), between 5 and 10% of all tumours had developed distant metastases during the 9-year follow-up time (Figure 3A and B). Among the tumours with high proliferative activity, between 25 and 30% of all tumours had developed distant metastases during the same time period (Figure 3A and B). However, when taking node status and tumour size into account, the role of proliferative activity as a risk factor becomes more evident (Figure 4A–D). In node-negative (N0) tumours smaller than 20 mm (T1) in which the proliferative activity was low, only 2–3% of the tumours developed distant metastases during the 9-year follow-up time (Figure 4A and C). For node-positive (N+), T1 tumours with low proliferative activity, the risk of developing distant metastases had increased slightly to between 5 and 7% (Figure 4B and D). In tumours equal to or larger than 20 mm (T2), the risk of developing distant metastasis had increased somewhat further in the node-negative (N0) tumour group to between 8 and 12% (Figure 4A and C), but substantially in the node-positive (N+) tumour group to between 30 and 40% (Figure 4C and D). In tumours of high proliferative activity, the risk of developing distant metastases was considerably increased in all tumour groups. For N0 tumours, the risk was between 20 and 25% (Figure 4A and C), and in N+ tumours it was between 35 and 55% (Figure 4B and D).

An increased risk of distant metastases was also seen in the highly aneuploid A-tumours as compared with the near-diploid D-tumours (Figure 4). However, this increased risk could only be demonstrated in small (T1), node-negative (N0) tumours, among which the D-tumours exhibited a risk as low as 2–3% and the A-tumours as high as 20–25% of developing distant metastases (Figure 4E). For the remaining tumour groups T2N0, T1N+ and T2N+, no significant difference between D- and A-tumours could be demonstrated (Figure 4E and F).

**Figure 5:**
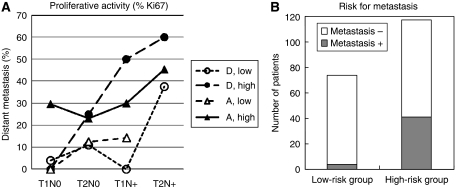
Relative risk of developing distant metastases in patients with T1N0 T2N0, T1N+ and T2N+ tumours of D- and A-tumour type showing low and high proliferative activity using 15% Ki67 positive cells as cut off value (**A**). By combining ploidy type, proliferative activity, node status and tumour size two breast cancer risk groups could be identified with respect to developing distant metastasis (**B**).

The relative contributions of genomic instability and proliferative activity as risk factors for distant metastases became very evident when the tumours were divided into four groups, D-tumours with low proliferative activity (D low), D-tumours with high proliferative activity (D high), A-tumours with low proliferative activity (A low) and A-tumours with high proliferative activity (A high), and also taking node status and tumour size into account at the same time (Table 1 and Figure 5A). In the group T1N0, all D-tumours, independently of proliferative activity, showed a very low risk (less than 3%) of developing distant metastases (Table 1 and Figure 5A). For the A-tumours in the group T1N0, the situation was different. A-tumours with low proliferative activity, however, showed the same low risk of developing distant metastases as the D-tumours, but for the A-tumours with high proliferative activity, the risk of developing distant metastases was found to be high (close to 30%) and significantly increased (*P*<0.01) over that of the D-tumours with high proliferative activity. This means that the A-tumours with high proliferative activity metastasize very early, contrary to D-tumours with high proliferative activity, and that the genomic instability associated with the ploidy type A adds prognostic information in addition to that of proliferative activity *per se*. For large (T2) node-negative (N0) tumours, or small (T1) node-positive (N+) tumours, the risk of distant metastases was only moderately increased for tumours with low proliferative activity, 0–12% for the D-tumours and 13–17% for the A-tumours (Table 1), again indicating that ploidy gives information in addition to that of proliferative activity. Large (T2) node-positive (N+) tumours were at high risk of metastasis relatively independent of ploidy type or proliferative activity (Figure 5C and Table 1).

By combining ploidy type (D or A), proliferative activity (low or high), node-status (N0 or N+) and tumour size (T1 or T2), breast cancers could be divided into two risk groups with respect to development of distant metastases (Figure 5D). For the low-risk group, consisting of all D-tumours in the T1N0 group together with the A-tumours with low proliferative activity in the same group plus all tumours with low proliferative activity in the T2N0 and T1N+ groups, the risk of developing distant metastases was less than 6%. For the high-risk group, consisting of all large (T2) node-positive (N+) tumours and all tumours with high proliferative activity, except the D-tumours in the T1N0 group, the risk of distant metastases was found to be in the range 30–60% (Figure 5C) and around 35% in average (Figure 5D). The low-risk group defined in this way is relatively large and constitutes about 40% of all women with stage T1 and T2 breast cancer (Figure 5B). By defining the low-risk group in a more stringent way, only taking into account all the D-tumours in the T1N0 group plus the A-tumours with low proliferative activity in the same group, the low-risk group is still relatively large and now constitutes around 25% of all women with breast cancer. The risk of developing distant metastases in this more stringently defined group is now as low as around 2% (Table 1). Multivariate analysis showed that proliferation (both Ki67 and cyclin A) was the most critical factor in N+ tumours (*P*<0.05) and ploidy was the most critical one in N0 tumours (*P*<0.05).

## Discussion

In this study, it was found that both genomic instability and proliferative activity can influence risk of developing distant metastases independently of each other, and that they have different relative impacts depending on tumour size and node status. One important methodological aspect of the approach used in this paper is that the Ki67 and the cyclin A analyses were carried out on identical microscopic areas of each tumour. This is particularly important when considering the large variations in proliferative activity that was found to exist between different areas in the tumours. In addition to obtaining accurate information about proliferative activity by using two independent markers analysed in several identical microscopic areas of each tumour, a direct quantitative comparison between the use of Ki67 and cyclin A could also be performed in an accurate way. A second important methodological aspect of this paper is that proliferative activity and genomic instability could be studied simultaneously as independent risk factors in the same individual tumour. This gives particular strength to the quantitative approach of this study.

The frequency of cyclin A-positive cells was highly correlated to the frequency of Ki67 cells, both in the analysis of each individual microscopic area and in the whole set of the 428 tumours. This clearly shows that both Ki67 and cyclin A are equally reliable as markers of proliferative activity. The average frequency value of the cyclin A-stained cells was found to be about 0.4 times that of the corresponding Ki67 values. As Ki67 is expressed throughout most of the cell cycle (Gerdes, 1990), and the expression of cyclin A is restricted to the S- and G2-phases in both normal and tumour cells (Erlandsson *et al*, 2000), the data indicate that in breast tumours the S- and G2-phases on average occupies about 40% of the whole cell cycle as defined by Ki67.

A lower but still relatively good correlation was also found between Ki67 and SPF and between cyclin A and SPF. The true correlation is likely to be much higher taking into account the large variation in proliferative activity seen between different microscopic fields and the fact that the SPF was obtained from cytophotometric measurements of only about 100 cells from just a few microscopic fields in each tumour. Another factor that tends to lower the correlation between SPF and Ki67 or cyclin A is the overrepresentation of normal cells measured in the S-phase analysis. This risk is, however, relatively low when SPF is based on image cytometric analysis, in which only morphologically identified cells are measured. For flow cytometric analysis, this error in determining the SPF can be substantial, particularly in the near-diploid tumours where all cells in the tumour are measured without any possibility to make a discrimination between normal and tumour cells on the basis of DNA content. For the clearly aneuploid tumours, the admixture of normal cells in the tissue sample can to some extent be estimated from the co-existence of cells with diploid DNA values. However, a problem with the clearly aneuploid tumours on the other hand is that some of the cells with DNA values in the S-phase region may in fact represent non-proliferating or growth-arrested cells with numerical chromosomal aberrations. It is thus of decisive importance to be aware of these difficulties when calculating SPFs from flow cytometry data (Falkmer *et al*, 1990). In spite of these methodological deficiencies, it has been shown that high SPF is a property that is related to poor prognosis for many tumours (Kallioniemi *et al*, 1987).

In contrast to the interpretation of DNA histograms based on flow-cytometric measurements, image cytometry based on DNA histograms were preferentially interpreted by subdividing the histograms in diploid or pseudo-diploid (D-tumours) and highly aneuploid types (A-tumours) (Zetterberg and Esposti, 1980; Cornelisse *et al*, 1987; Fallenius *et al*, 1988a, 1988b; Forsslund and Zetterberg, 1990; Forsslund *et al*, 1996). The large difference in the extent of aneuploidy that exists between the highly aneuploid A-tumours on one hand and the pseudo-diploid D-tumours on the other is most likely generated by two principally different mechanisms. Evidence has been obtained that a defective coordination between the centrosome cycle and the DNA replication cycle leading to abnormal, often tripolar, mitoses and highly unequal segregation of chromosomes between daughter cells is crucial for the generation of high-degree aneuploidy seen in the A-tumours (Kronenwett *et al*, 2005). The low-degree aneuploidy seen in the pseudo-diploid D-tumours on the other hand is more likely to be generated by some defect primarily in the chromatid segregation mechanism at mitosis.

One of the main points of this paper was to quantitate the role of genomic instability on one hand and proliferative activity on the other as independent risk factors for the development of distant metastases. The relative contributions of each of these two risk factors became clearly evident first when the tumours were divided into different subgroups with respect to ploidy type and proliferative activity (D low, D high, A low and A high), and also taking node status and tumour size into account at the same time. Although increased proliferative activity in general was associated with an increased risk of developing distant metastases, multivariate analysis showed that ploidy level was an independent and even stronger marker when considering small (T1) node-negative (N0) tumours. In this group (T1N0), all D-tumours, independently of proliferative activity, showed a very low risk of developing distant metastases. This was also found for grade 1 tumours, although histological grade in general was found to have less impact as risk marker in comparison with proliferative activity and ploidy. For the A-tumours in the same group (T1N0), the situation was different. A-tumours with low proliferative activity showed about the same low risk of developing distant metastasis as the D-tumours, but for the group of A-tumours with high proliferative activity, the risk of developing distant metastases was found to be high and significantly increased over that of the D-tumours with high proliferative activity. This means that the A-tumours with high proliferative activity metastasise very early, contrary to D-tumours with high proliferative activity. Thus the genomic instability associated with the ploidy type A adds prognostic information that is independent of the proliferative activity *per se*. Genomic instability is therefore likely to be a crucial property in the process of forming distant metastases, and aneuploidy *per se* as one mechanism of generating gene copy number imbalances in the tumour is one important aspect of genomic instability. This is further supported by our previous findings that chromosomal rearrangements in terms of deletions, duplications and amplifications, as studied by CGH, were much more frequent in the highly aneuploid breast tumours than in the pseudo-diploid ones (Blegen *et al*, 2001). With high-resolution microarray-based CGH, we could furthermore show that overall the degree of chromosomal rearrangements as well as specific patterns of rearrangements were both related to aggressive tumour behaviour (Hicks *et al*, 2006). Collectively, these data clearly indicate that genomic instability is an important factor for tumour progression and metastasis.

The possibility to classify breast tumours accurately into risk groups on the basis of genomic instability together with information about proliferation is an implication of practical clinical relevance of this study. It is clear from the findings in this paper that by combining ploidy type, proliferative activity and tumour stage, a large group of low-risk breast tumours could be identified, in which only a few percentage of the tumours developed distant metastases. It is obvious that it would have great practical implications when selecting patients for different therapy regimes if risk of distant metastases could be predicted objectively with high degree of precision.

## Figures and Tables

**Table 1:** Number and percentage of node-negative (N0) and node-positive (N+) T1 and T2 breast carcinomas with low or high proliferative activity (Ki67/cyclin A) belonging to the D- or A-tumour type

	**Tumour size in N0 tumours**		**Tumour size in N+ tumours**	
	**T1 *n* (%)**	**T2 *n* (%)**	**T1 *n* (%)**	**T2 *n* (%)**
*Proliferation*				
Low (Ki67)	1/35 (2.86)	2/23 (8.70)	1/15 (6.67)	3/8 (37.50)
Low (cyclin A)	1/42 (2.38)	3/25 (12.00)	1/19 (5.26)	4/12 (33.33)
High (Ki67)	8/39 (20.51)	9/40 (22.50)	6/17 (35.29)	19/36 (52.78)
High (cyclin A)	8/32 (25.00)	8/38 (21.05)	6/13 (46.15)	18/32 (56.25)
				
*Ploidy*				
D	1/36 (2.78)	3/17 (17.65)	3/12 (25.00)	9/18 (50.00)
A	8/35 (22.86)	7/34 (20.59)	4/17 (23.53)	10/22 (45.45)
				
*Ploidy*+*proliferation*				
D+low (Ki67)	1/25 (4.00)	1/9 (11.11)	0/6 (0)	3/8 (37.50)
D+low (cyclin A)	1/32 (3.13)	1/8 (12.50)	0/9 (0)	2/8 (25.00)
D+high (Ki67)	0/11 (0)	2/8 (25.00)	3/6 (50.00)	6/10 (60.00)
D+high (cyclin A)	0/4 (0)	2/9 (22.22)	3/3 (100.00)	7/10 (70.00)
A+low (Ki67)	0/8 (0)	1/8 (12.50)	1/7 (14.29)	0/0
A+low (cyclin A)	0/7 (0)	2/10 (20.00)	1/8 (12.50)	2/4 (50.00)
A+high (Ki67)	8/27 (29.63)	6/26 (23.08)	3/10 (30.00)	10/22 (45.45)
A+high (cyclin A)	8/28 (28.57)	5/24 (20.83%)	3/9 (33.33%)	8/18 (44.44%)
